# RETRACTION: An Evaluation of the Effectiveness of ‘ULTRACOL 200′ in Enhancing Nasolabial Fold Wrinkles Through Cutaneous Repair

**DOI:** 10.1111/srt.70286

**Published:** 2025-10-29

**RETRACTION**: N. N. Giang, H. Kim, P. N. Chien, H. J. Kwon, J. R. Ham, W. K. Lee, Y. J. Gu, S. Y. Zhou, X. R. Zhang, S. Y. Nam, and C. Y. Heo, “An Evaluation of the Effectiveness of ‘ULTRACOL 200′ in Enhancing Nasolabial Fold Wrinkles Through Cutaneous Repair,” *Skin Research and Technology *30, no. 4 (2024): e13679, https://doi.org/10.1111/srt.13679.

The above article, published online on 14 April 2024 in Wiley Online Library (wileyonlinelibrary.com), has been retracted by agreement between *Skin Research and Technology*; and John Wiley & Sons Ltd. The article was accepted for publication following an insufficient peer review process that does not meet the standards of the journal's peer review policy. Furthermore, the article lacks critical methodological details necessary for reproducing and interpreting the findings. It also fails to disclose relevant financial and competing interests, despite several co‐authors being employed by the manufacturer of the test product. Accordingly, the article has been retracted. The authors have been informed of the retraction decision. Authors Nguyen Ngan Giang, Xin Rui Zhang and Chan Yeong Heo disagreed with this decision; no confirmation was obtained by the remaining co‐authors.

